# Avaliação do conhecimento de pacientes diabéticos sobre medidas preventivas do pé diabético em Maringá (PR)

**DOI:** 10.1590/1677-5449.006416

**Published:** 2017

**Authors:** Guilherme Pereira Carlesso, Mariana Helena Barboza Gonçalves, Dorival Moreschi

**Affiliations:** 1 Centro Universitário Cesumar – UniCesumar, Cirurgia Vascular, Maringá, PR, Brasil.

**Keywords:** pé diabético, prevenção primária, conhecimento, diabetes

## Abstract

**Contexto:**

O atual envelhecimento da população tem gerado maior predominância de doenças crônicas, como o diabetes, a qual está associada a um risco elevado de complicações crônicas e agudas. Entre essas, o pé diabético (PD) destaca-se por possuir alta incidência e grande poder mutilador.

**Objetivo:**

Avaliar o conhecimento da população diabética das Unidades Básicas de Saúde (UBS) de Maringá (PR) sobre a prevenção do PD.

**Métodos:**

Estudo descritivo, quantitativo, tipo inquérito por entrevista. A população estudada foi composta por 80 portadores de diabetes, cadastrados em UBS de Maringá (PR). A coleta de dados buscou levantar dados sociodemográficos e epidemiológicos, bem como as atitudes de controle do diabetes e do autocuidado para prevenção do PD.

**Resultados:**

Do total de entrevistados, nove não realizavam qualquer tipo de exame para controle do diabetes e a renda mensal predominante foi de até um salário mínimo. O grau de escolaridade e a renda mensal não se mostraram relevantes em relação ao conhecimento de cuidados preventivos do PD e nem uma maior adesão a hábitos de vida saudáveis. O cuidado com o PD tende a melhorar à medida que exista uma compreensão mais clara dos fatores que conduzem à perda do membro e um crescente consenso sobre a gestão de vários aspectos clínicos do cuidado com o pé.

**Conclusão:**

Existe uma falta de aprendizado das medidas preventivas, mesmo nos pacientes com algum nível de instrução, o que induz a uma prática deficiente de cuidados.

## INTRODUÇÃO

Com o envelhecimento da população brasileira, houve uma inversão do perfil epidemiológico, com o predomínio de condições crônicas em detrimento das doenças infectocontagiosas^1^. O diabetes é uma condição de alta morbidade que afeta mais de 220 milhões de pessoas no mundo, sendo estimado 336 milhões para 2030^2^, e está associado a um alto risco de desenvolvimento de complicações agudas e crônicas. Entre essas morbidades, o pé diabético é a que mais se destaca na área da cirurgia vascular, por possuir aspecto mutilador, levando frequentemente à amputação, em especial quando a osteomielite e a infecção da ferida estão presentes^3^.

O pé diabético é definido como infecção, ulceração e/ou destruição dos tecidos profundos associadas a anormalidades neurológicas e doença vascular periférica nos membros inferiores^4^, com incidência de 15% dos pacientes diabéticos nos EUA^5^. O pé diabético é uma condição que engloba diversas patologias, como a própria neuropatia, a doença arterial periférica e a ulceração do pé, além da neuroartropatia de Charcot e a osteomielite^6^.

A maioria das internações é decorrente das úlceras nos diabéticos^3^, as quais são a principal complicação da doença, acometendo principalmente membros inferiores^7^. As ulcerações chegam a afetar 15% dos diabéticos em países desenvolvidos, sendo responsáveis por 6 a 20% das hospitalizações^8^, ressaltando-se que cerca de 85% das amputações são precedidas por úlcera^3^. O Consenso Internacional de Pé Diabético é categórico ao afirmar o significativo problema socioeconômico que o pé diabético causa, tanto em relação aos gastos para internação e amputação para os sistemas de saúde, quanto para o paciente, que enfrenta perda de produtividade e de qualidade de vida, adicionada aos custos individuais de cada um.

Para a prevenção das internações e diminuição dos riscos de amputação, uma atenção básica orientada e capacitada é eficaz na vigilância e controle da doença, constituindo também uma importante fonte de coleta de dados^8^. Para isso, o profissional deve ser treinado para rastreio e diagnóstico, além de ser habilitado a instruir o paciente ao autocuidado, como o uso de calçados adequados e maneiras corretas de cortar de unhas. No rastreamento, a busca por fatores de risco, como o mau controle da hemoglobina glicada e glicemia de jejum, história de úlcera prévia, conhecimento precário quanto ao diabetes e problemas nos pés são bastante relevantes para esse tipo de abordagem^3^.

Pacientes que apresentam sintomas neuropáticos e vasculares (como claudicação intermitente) associados a fatores de risco para complicações, como tabagismo e descontrole glicêmico, merecem atenção especial pelo profissional de saúde. A instrução quanto ao autoexame dos pés diariamente e o cuidado especial com os pés devem ser abordados firmemente durante as consultas com os pacientes diabéticos, levando-os a um estado de constante observação e alerta quanto às manifestações clínicas que podem evoluir para neuropatia ou úlcera diabética^9^.

Um estudo feito acerca da representação social dos termos “diabetes” e “pé diabético” mostrou uma falta de comunicação e interação entre os profissionais da saúde e os diabéticos nas rotinas de atendimento, sendo que os primeiros mostraram-se preocupados tecnicamente a respeito de seus serviços prestados ao paciente diabético, indicando a necessidade de programas de conscientização e orientação ao autocuidado em diabetes^10^.

O objetivo deste artigo foi avaliar o conhecimento de pacientes diabéticos atendidos nas Unidades Básicas de Saúde (UBS) de Maringá-(PR) sobre a prevenção do pé diabético e traçar um perfil socioeconômico-cultural da amostra, relacionando as variáveis estudadas com o nível de conhecimento dessa complicação do diabetes.

## METODOLOGIA

Trata-se de um estudo descritivo, quantitativo, tipo inquérito por entrevista com amostra selecionada no munício de Maringá (PR). Para o presente estudo, foram entrevistados 80 portadores de diabetes, com idade mínima de 30 anos. A coleta de dados foi feita através dos Núcleos Integrados de Saúde (NIS) Cidade Alta, Tuiuti e Alvorada, do município de Maringá (PR), por meio do acompanhamento dos grupos de Hipertensos e Diabéticos (HIPERDIA) de cada Programa de Saúde da Família (PSF) e através de busca ativa, realizando visita domiciliar com base nos dados fornecidos pelos NIS.

Todos os entrevistados aceitaram participar do estudo mediante leitura e assinatura do termo de consentimento livre e esclarecido, aprovado previamente pelo Comitê de Ética da UniCesumar – Centro Universitário Cesumar.

Por meio de questionário foram levantados dados sociodemográficos e epidemiológicos, bem como as atitudes de controle do diabetes e do autocuidado para prevenção do pé diabético. Após o questionário, os pesquisadores instruíram os entrevistados através de folder elucidativo contendo informações a respeito dos cuidados do pé diabético. O questionário foi desenvolvido baseado nos estudos de Cosson et al.^11^ e Bragança et al.^12^, os quais abordaram o pé diabético de maneira semelhante à nossa.

A análise estatística foi realizada através de descrições tabulares e gráficas do perfil da amostra e da frequência percentual obtida para cada uma das variáveis do estudo através do programa Microsoft Excel 2013, por meio de estatística descritiva simples e discutida à luz da literatura sobre o tema.

## RESULTADOS

Do total de entrevistados, 40% pertenciam ao sexo masculino e 60% ao feminino. A idade dos entrevistados variou entre 31 e 92 anos, com uma mediana de 69 e média de 68,7 anos, sendo que praticamente a metade deles possuía apenas ensino fundamental completo. A renda mensal predominante era de até um salário mínimo (41,3%).

O diagnóstico de diabetes tinha sido feito há mais de 4 anos em 75% dos pacientes. O número de fumantes em ambos os sexos se aproximou, somando cerca de 20% da amostra, sendo que todos fumavam há mais de 15 anos.

Dos 80 entrevistados, nove não realizavam qualquer tipo de exame para controle do diabetes, sendo seis do sexo feminino (Tabela 1).

**Tabela 1:** Número de entrevistados que realizam algum exame para controle da diabetes por sexo.

**Controle com exames**	**Feminino**	**Masculino**	**Total**
Sim	42	29	71
Não	6	3	9
Total	48	32	80

Quanto aos hábitos de vida (Tabela 2), poucos pacientes realizavam dieta para diabetes e praticavam atividade física regular; em contrapartida, quase metade dos entrevistados não seguiam dieta e nem rotina de exercícios físicos. Além disto, poucos (11,3%) faziam apenas exercícios e também poucos (17,5%) realizam somente dieta. Do total dos que faziam dieta, cerca de 84% seguiam a orientação médica para tal, representando apenas 35% do total de entrevistados. Dos que praticavam atividade física, mais da metade (57,1%) faziam-na mais de três vezes na semana.

**Tabela 2:** Relação entre a prática de hábitos de vida saudáveis e sexo.

**Hábitos de vida**	**Feminino**	**Masculino**	**Total**
Fazem dieta e exercício	11	8	19
Fazem dieta, mas não exercício	11	3	14
Fazem exercício, mas não dieta	4	5	9
Não fazem dieta e exercício	22	16	38

Cruzando-se os dados da escolaridade e da prática de hábitos saudáveis (dieta e exercício), concluiu-se que aqueles que realizavam ambas as recomendações ou que não realizavam nenhuma delas tinham a mesma escolaridade (Tabela 3). Além disso, a renda mensal não refletiu uma maior preocupação com hábitos de vida saudáveis, como mostrado na Tabela 4.

**Tabela 3:** Relação de hábitos de vida saudável com a escolaridade dos entrevistados.

**Hábitos de vida**	**SC**	**SI**	**MC**	**MI**	**FC**	**FI**	**A**
Fazem dieta e exercício	2	0	1	2	2	9	6
Fazem dieta, mas não exercício	2	0	3	1	1	6	1
Fazem exercício mas não dieta	0	1	0	0	1	3	4
Não fazem dieta e exercício	2	0	3	0	3	23	4

SC: superior completo; SI: superior incompleto; MC: médio completo; MI: médio incompleto; FC: fundamental completo; FI: fundamental incompleto; A: analfabeto.

**Tabela 4:** Relação entre hábitos de vida saudáveis com a renda mensal, em número de salários mínimos.

**Hábitos de vida**	**0-1 salário mínimo**	**1-2 salários mínimos**	**> 2 salários mínimos**
Fazem dieta e exercício	7	6	7
Fazem dieta, mas não exercício	5	5	4
Fazem exercício, mas não dieta	3	3	3
Não fazem dieta e exercício	19	11	7

Em relação aos cuidados com os pés, os números variaram entre as questões. Ao mesmo tempo em que 87,5% não tinham o hábito de andar descalço, sendo este um fator positivo, mais de 96% desconheciam os sapatos específicos para diabéticos. Quase todos os pacientes referiram ter a pele dos pés ressecada. Na Tabela 5 podem ser vistos outros dados em relação a esses cuidados. Ao relacionarmos os questionamentos referentes ao cuidado com os pés e a escolaridade, concluiu-se que os dados não apresentavam nenhuma correlação.

**Tabela 5:** Número de entrevistados e suas respostas quanto ao conhecimento de medidas preventivas do pé diabético.

**Cuidados preventivos ao pé diabético**	**Sim (%)**	**Não (%)**
Hábito de andar descalço	10 (12,5%)	70 (87,5%)
Uso de sapatos fechados com meia	45 (56,3%)	35 (43,7%)
Hábito de hidratar os pés com cremes ou óleos	45 (56,3%)	35 (43,7%)
Corta unhas de forma adequada	59 (73,7%)	21 (26,3%)
Apresentação de micose interdigital	8 (10%)	72 (90%)
Presença de rachaduras	13 (16,2%)	67 (83,8%)
Presença de pele ressecada dos pés	55 (68,7%)	25 (31,3%)
Presença de calos nos pés	9 (11,3%)	71 (88,7%)
Uso de sapatos próprios para diabéticos	3 (3,7%)	77 (96,3%)

Outras conclusões a serem retiradas dessa relação compreendem que o número de mulheres que possui o hábito de hidratar os pés foi quatro vezes maior quando comparado ao dos homens; em contrapartida, o dobro de mulheres cortava as unhas de maneira inadequada e a maioria não usava meias com sapatos fechados.

## DISCUSSÃO

A Organização Mundial da Saúde define o diabetes como uma doença crônica caracterizada por níveis constantemente elevados de glicemia (maior que 126 mg/dL) resultante de defeitos na secreção e ação da insulina. Essa característica leva a complicações micro e macrovasculares, que abrangem retinopatia, nefropatia, doença arterial periférica e lesões ulcerativas de membros inferiores, conhecidas como síndrome do pé diabético^13^.

Define-se por pé diabético a presença de infecção, ulceração e/ou destruição de tecidos profundos associada a anormalidades neurológicas e doença vascular periférica^6^. A doença neurológica é um importante fator de risco para o desenvolvimento de ulceração. A neuropatia sensório-motora periférica e a neuropatia autonômica estão entre as formas mais comuns de manifestação neurológica^14^^,^ 15.

A amostra deste estudo foi aleatória, pois não tínhamos na literatura uma base do número de pacientes a serem entrevistados. Além disso, apenas uma pequena porcentagem de pacientes diabéticos participa dos grupos de pacientes que se reúnem periodicamente nas UBS para avaliações e instruções.

Em um estudo realizado com 109 pessoas em Rio Branco (AC), o grau de escolaridade não se mostrou relevante em relação ao conhecimento de cuidados preventivos do pé diabético^11^, provavelmente porque, mesmo com grau de escolaridade maior, os pacientes não têm acesso a informações sobre as neuropatias e vasculopatia.

As alterações de sensibilidade à dor são prejudiciais, pois esta é uma condição protetora frente a traumas ou desconfortos que poderiam culminar em lesão^15^^,^ 16. Além disso, muitos médicos não compreendem como pacientes com alto grau de escolaridade chegam aos consultórios de cirurgia vascular com úlcera induzida por sapatos de tamanho inapropriado. Isso pode ser explicado pelo fato de muitos pacientes já apresentarem algum grau de neuropatia, e sapatos de menor tamanho acabam estimulando o resquício de sensibilidade ali presente, possibilitando a interpretação de que o sapato está de tamanho adequado^15^. Logo, não se trata de um problema de cunho intelectual, mas sim de lesão nervosa.

O questionamento sobre os hábitos de andar descalço e utilizar meias com sapatos fechados teve o objetivo de identificar hábitos de risco para o desenvolvimento de ulceração. Um estudo feito na cidade de Campinas mostrou porcentagens semelhantes quanto ao uso de sapatos com meia; porém, o hábito de andar descalço mostrou-se mais frequente entre os campinenses (36%)^12^ que entre os maringaenses, analisados em nosso estudo (12,5%). Contudo, pela considerável porcentagem de analfabetos (18,8%), o estudo campinense chama atenção para a criação de estratégias educacionais diferenciadas para atender essa população, de maneira que facilite o autocuidado^12^.

Em relação ao uso de sapatos fechados com meia, obtivemos 43,8% de resposta afirmativa, ficando na média de outros dois estudos que encontraram valores de 57% e 37,5%, respectivamente^12^^,^ 13. A qualidade das meias é de suma importância, devendo ser preferencialmente de lã ou algodão, sem costuras e que sejam trocadas uma vez ao dia, evitando, desse modo, traumas causados pelo sapato^17^.

Os calçados adequados são aqueles que suportam e protegem os pés contra traumas mecânicos, distribuindo os pontos de pressão, que não apresentam costuras e que estão em bom estado de conservação. Os sapatos não devem ser muito largos e nem muito apertados, pois esses tipos de calçado favorecem o atrito e aparecimento de bolhas, e o ideal é que sejam adquiridos no período da tarde, momento em que os pés tendem a estar edemaciados^17^. Nosso estudo apontou que quase todos os entrevistados tinham desconhecimento dessas características, sendo priorizado apenas o conforto na hora da compra.

Apesar de a escolaridade não estar estritamente relacionada com úlcera diabética, outros fatores como deficiência visual, falta de equilíbrio, diminuição da flexibilidade dos membros atuam como limitantes da capacidade de reconhecer anormalidades dos pés^18^.

O pé diabético relaciona-se com o tempo de duração do diabetes e com a idade do paciente^18^. Ao longo da vida, a incidência de úlcera nos pés em pacientes diabéticos é estimada entre 12 e 25%^19^. Estima-se que mais de 50% de pacientes mais velhos com diabetes tipo 2 possuam alguma evidência de perda sensorial no exame clínico, um fator de risco para ulceração, e que 13% dos pacientes possuam perda sensorial importante no momento do diagnóstico de diabetes^15^. Esses números mostram que o exame periódico dos pés em pacientes diabéticos de qualquer idade, principalmente em atenção primária, fariam a detecção precoce de alterações neuropáticas relevantes para reforço de condutas terapêuticas e informações sobre o autocuidado.

Nos estudos de Santos et al.^16^ e Gamba et al.^20^, a maioria das pessoas submetidas a amputação possuíam mau controle metabólico, não tinham acesso à informação de cuidados preventivos, não aderiram ao tratamento clínico e apresentavam dificuldades financeiras. Ademais, a amputação e a perda do membro têm impacto maior que qualquer outra complicação do diabetes, pois, além da perda da mobilidade e da independência, são frequentes a ansiedade e a depressão^21^^,^ 22.

O tabagismo é um cofator agravante que acelera o processo de aterosclerose em artérias tibiais^23^. Este, somado ao trauma, calçados impróprios, corpos estranhos nos pés e cortes inadequados nas unhas, contribui para o aumento da frequência de pé diabético^11^. Esse fator deve ser valorizado, pois, no presente estudo, cerca de 20% da amostra mantêm o hábito de fumar, ainda que conscientes do seu risco.

Os cuidados com o pé diabético melhoram à medida que se tenha uma compreensão mais clara dos fatores que conduzem à perda do membro e um crescente consenso sobre os vários aspectos que devem ser tomados em relação ao pé^4^^,^ 24.

Os programas desenvolvidos pelo Ministério da Saúde em atenção aos portadores de diabetes têm por pressuposto que o seguimento de rotina dos pacientes seja feito pelo eixo estruturante das UBS e pela Estratégia de Saúde da Família (ESF)^16^. Porém, não existem avaliações do resultado desses programas referentes às medidas de prevenção do pé diabético.

Este estudo observou que existem lacunas com relação aos grupos de atenção básica voltados à orientação e abordagem dos hipertensos e diabéticos. Esses grupos, denominados grupos HIPERDIA, mostraram-se aparentemente insatisfatórios quanto às recomendações impostas pelo Ministério da Saúde, descumprindo o propósito principal da atenção básica, isto é, monitorar de forma contínua os pacientes com hipertensão e diabetes, tanto seus agravos quanto seus fatores de risco^25^.

É de competência do enfermeiro a orientação sobre mudanças de estilo de vida e avaliação do potencial para o autocuidado, além de abordar outros fatores de risco, como condição socioeconômica e grau de escolaridade^25^. Entretanto, um estudo realizado na cidade de Caxias (MA) concluiu que o agente comunitário de saúde estabelece um vínculo mais efetivo e mais atuante na realização de orientações^26^, fato que subjetivamente também foi percebido pelos autores deste estudo.

Acreditamos que seria atribuição do Ministério da Saúde a elaboração de medidas educativas, tais como visitas e folhetos, voltadas ao conhecimento da população diabética, pois um controle glicêmico adequado, associado a hábitos de vida saudáveis e consultas periódicas voltadas a essa clientela, poderão reduzir a incidência de complicações. No entanto, faltam dados da literatura que demonstrem que essas medidas seriam efetivas nas complicações provenientes do chamando pé diabético.

Apesar de alguns estudos abordados em uma revisão sistemática do Instituto Cochrane concluírem que um maior conhecimento de medidas preventivas não altera a incidência de pé diabético, a mesma revisão conclui que as evidências desses estudos são precárias e com metodologia insuficiente para excluir da prática as recomendações aos pacientes com diabetes^27^.

Após as entrevistas, alertamos os pacientes do estudo sobre maneiras de autocuidado e dicas de prevenção através de folhetos educativos e abordagem individual, visando contribuir para uma diminuição das complicações dessa doença.

## CONCLUSÕES

Existe uma falta de conhecimento de medidas preventivas, mesmo nos pacientes com algum nível de instrução, em relação às possíveis complicações nos pés de pacientes diabéticos.

Uma renda mensal maior também não refletiu em maior interesse por hábitos de vida saudável, levando a crer que o autocuidado também é negligenciado.
