# Supplementary material for: Avaliação do conhecimento de pacientes diabéticos sobre medidas preventivas do pé diabético em Maringá (PR)
Source: J Vasc Bras. 2017 Apr-Jun;16(2):113–8. [Article in Portuguese] doi: 10.1590/1677-5449.006416 (PMC5915859; doi:10.1590/1677-5449.006416)
Supplement: Translation

# Translation: Evaluation of diabetic patients’ knowledge about preventive care of the diabetic foot, in Maringá, PR, Brazil

**Authors:** Guilherme Pereira Carlesso, Mariana Helena Barboza Gonçalves, Dorival Moreschi

**Affiliations:** 1 Centro Universitário Cesumar – UniCesumar, Cirurgia Vascular, Maringá, PR, Brazil.

**Keywords:** diabetic foot, primary prevention, knowledge, diabetes

## Abstract

**Background:**

One of the consequences of the current phenomenon of population aging is an increasing predominance of chronic diseases, such as diabetes, which is associated with a high risk of chronic and acute complications. Diabetic foot (DF) is of particular concern because of its high incidence and significant potential to mutilate.

**Objective:**

To assess knowledge about DF prevention in the diabetic population of primary care health centers (PHCC) in Maringá, PR, Brazil.

**Methods:**

This was a descriptive, quantitative study conducted by interview survey. The study population comprised 80 patients with diabetes registered at PHCCs in Maringá, PR, Brazil. Data collection encompassed sociodemographic and epidemiological data, and behavior related to diabetes control and self-care for DF prevention.

**Results:**

Nine of the interviewees did not take any type of test or undergo any examinations for diabetes control. The predominant monthly income bracket was less than the minimum wage. Neither educational level nor monthly income were relevant to knowledge about preventative care for DF or to better compliance with healthy lifestyle habits. Care of DF tends to improve to the extent that there is a clearer understanding of the factors that lead to limb loss and an increase in consensus on managing the various different clinical features of caring for the feet.

**Conclusions:**

There is a lack of knowledge about preventative measures, even among patients who have a reasonable level of education, resulting in deficient self-care behavior.

## INTRODUCTION

As the Brazilian population ages, its epidemiological profile is inverting, with chronic conditions taking over the predominant position previously held by infectious and contagious diseases.^1^ Diabetes is a condition that causes considerable morbidity and affects more than 220 million people worldwide and estimates for 2030 put the number at 336 million.^2^ It is associated with an elevated risk of development of both acute and chronic complications. and the diabetic foot is the diabetes complication with the greatest impact within vascular surgery, because of its propensity to mutilate, frequently causing amputations, particularly when osteomyelitis and wound infections are present.^3^

The diabetic foot is defined as infection, ulceration and/or destruction of deep tissues associated with neurological abnormalities and peripheral vascular disease in the lower limbs,^4^ with an incidence of 15% among diabetic patients in the United States.^5^ The diabetic foot is a condition that encompasses multiple pathologies, such as neuropathy, peripheral arterial disease, and ulceration of the foot, in addition to Charcot neuroarthropathy, and osteomyelitis.^6^

The majority of hospital admissions of diabetics are for ulcers,^3^ which are the major complication of the disease and primarily involve the lower limbs.^7^ Up to 15% of diabetics in developed countries develop ulcerations and they are responsible for 6 to 20% of hospital admissions,^8^ while around 85% of amputations are preceded by ulcers.^3^ The international diabetic foot consensus is categorical about the significance of the socioeconomic problems caused by the diabetic foot, both in terms of the costs to healthcare systems of admissions and amputations, and in terms of the patients’ lost productivity and quality of life, in addition to the individual expenditure they incur.

In order to prevent admissions and reduce the risks of amputation, well-managed primary care services provided by well-trained professionals are effective for surveillance and control of the disease, and also offer an important source for data collection.^8^ To achieve this, professionals must be trained for screening and diagnosis, and instructed on how to teach patients to care for themselves, providing information such as the appropriate footwear and the correct way to cut toenails. For monitoring, checking for risk factors such as poor control of glycated hemoglobin and fasting glycemia, previous history of ulcers, and poor knowledge about diabetes and problems with the feet is highly relevant to this type of approach.^3^

Patients who have neuropathic and vascular symptoms (such as intermittent claudication) combined with risk factors for complications, such as smoking and poor glycemic control, merit special attention from healthcare professionals. Instructions on daily foot self-examination and special care for feet should be covered robustly during consultations with diabetic patients, encouraging them to adopt a state of constant observation and alertness to clinical manifestations that could progress to neuropathy or diabetic ulcers.^9^

A study investigating the social representation of the terms “diabetes” and “diabetic foot” found that there was a lack of communication and interaction between health professionals and diabetics during routine care and that professionals were concerned with the technical aspects of the services they provided to diabetic patients, indicating that there is a need for awareness-raising and guidance about self-care in diabetes.^10^

The objectives of this study were to evaluate the knowledge of diabetic patients treated at primary care health centers (PHCC) in Maringá, PR, Brazil, about prevention of the diabetic foot and to trace a socioeconomic and cultural profile of the sample, relating the variables studied with their level of knowledge about this complication of diabetes.

## METHODOLOGY

This was a descriptive, quantitative study conducted by interview survey of a study sample comprising 80 patients selected from the population with diabetes registered at 3 PHCCs in Maringá, PR, Brazil. For the study, 80 patients with diabetes were interviewed, all over the age of 30. Data collection was conducted through the Integrated Health Centers (IHC) in Cidade Alta, Tuiuti, and Alvorada, in the municipal district of Maringá, PR, Brazil, as part of care for patients registered with the Hypertensive and Diabetic (HIPERDIA) programs at each Family Health Program (PSF) and by actively seeking patients during home visits based on data provided by the IHC.

All interviewees agreed to take part in the study and read and signed a free and informed consent form that had been approved in advance by the Ethics Committee at UniCesumar – Centro Universitário Cesumar.

A questionnaire was used to collect sociodemographic and epidemiological data and to ask about behavior related to control of diabetes and self-care for prevention of diabetic foot. After administration of the questionnaire, the researchers used an educational folder containing information on care for the diabetic foot to teach the interviewees. The questionnaire was developed based on work by Cosson et al.^11^ and Bragança et al.,^12^ who studied the diabetic foot in a similar study to ours.

Statistical analysis comprised tables and graphs to illustrate the profile of the sample and the percentage frequencies for each of the study variables, using Microsoft Excel 2013, and calculation of simple descriptive statistics and discussion of their implications in relation to the literature on the subject.

## RESULTS

Of the total sample of 80 interviewees, 40% were male and 60% were female. Interviewees’ ages ranged from 31 to 92 years, with a median of 69 and a mean of 68.7 years. Almost half of the sample had only completed primary education. The most common monthly income category was below the minimum wage (41.3%).

Diabetes had been diagnosed more than 4 years previously in 75% of the patients. The number of smokers was similar across the two sexes and was around 20% of the sample, all of whom had smoked for more than 15 years.

Nine of the 80 interviewees, six of whom were women, did not take any type of test or undergo any examinations for diabetes control (Table 1).

**Table 1:** Number of interviewees who undergo tests or examinations for diabetes control, by sex.

**Control tests/examinations**	**Female**	**Male**	**Total**
Yes	42	29	71
No	6	3	9
Total	48	32	80

With regard to lifestyle habits (Table 2), few patients followed a diet for diabetics and engaged in physical activity regularly; whereas almost half of the interviewees did not follow the diet or routinely perform physical exercises. Additionally, a small percentage (11.3%) only did exercise and a small percentage (17.5%) only followed a diet. Around 84% of those who did keep to a diet were following medical guidance, although this was still only 35% of the total sample of interviewees. More than half of those who engaged in physical activity, (57.1%) did so more than three times a week.

**Table 2:** Relationship between healthy lifestyle habits and sex.

**Lifestyle habits**	**Female**	**Male**	**Total**
Follows diet and exercises	11	8	19
Follows diet, but does not exercise	11	3	14
Exercises, but does not follow diet	4	5	9
Does not follow diet and does not exercise	22	16	38

Analysis of data on educational level against healthy lifestyle habits (diet and exercise) showed that those who followed both recommendations and those who did not follow either had the same educational level (Table 3). Additionally, monthly income was not reflected in greater concern for healthy lifestyle habits, as shown in Table 4.

**Table 3:** Relationship between healthy lifestyle habits and educational level of interviewees.

**Lifestyle habits**	**HEC**	**HES**	**SEC**	**SES**	**PEC**	**PES**	**IL**
Follows diet and exercises	2	0	1	2	2	9	6
Follows diet, but does not exercise	2	0	3	1	1	6	1
Exercises but does not follow diet	0	1	0	0	1	3	4
Does not follow diet and does not exercise	2	0	3	0	3	23	4

HEC: Higher education, completed; HES: Higher education, started; SEC: Secondary education, completed; SES: Secondary education, started; PEC: Primary education, completed; PES: Primary education, started; IL: Illiterate.

**Table 4:** Relationship between healthy lifestyle habits and monthly income, in multiples of minimum monthly wage.

**Lifestyle habits**	**0-1 times minimum wage**	**1-2 times minimum wage**	**> 2 times minimum wage**
Follows diet and exercise	7	6	7
Follows diet, but does not exercise	5	5	4
Exercises but does not follow diet	3	3	3
Does not follow diet and does not exercise	19	11	7

The results for care for the feet varied across different questions. While 87.5% were not in the habit of going barefoot, which is a positive factor, more than 96% did not know about shoes specifically for diabetics. Almost all of the patients reported having dry skin on their feet. Table 5 lists the results for these and other variables related to caring for the feet. Analysis of the answers to questions about foot care against educational level showed that the data were not correlated.

**Table 5:** Number of interviewees and their replies to questions about their knowledge about preventative measures for the diabetic foot.

**Preventative care for the diabetic foot**	**Yes (%)**	**No (%)**
Goes barefoot	10 (12.5%)	70 (87.5%)
Wears socks with closed shoes	45 (56.3%)	35 (43.7%)
Moisturizes feet with creams or oils	45 (56.3%)	35 (43.7%)
Cuts toenails correctly	59 (73.7%)	21 (26.3%)
Has mycosis between toes	8 (10%)	72 (90%)
Has cracked skin	13 (16.2%)	67 (83.8%)
Has dried skin on feet	55 (68.7%)	25 (31.3%)
Has corns on feet	9 (11.3%)	71 (88.7%)
Wears shoes appropriate for diabetics	3 (3.7%)	77 (96.3%)

Other conclusions can be drawn with relation to these measures. Four times as many women as men used moisturizer on their feet; whereas twice as many women cut their toenails incorrectly and the majority did not wear socks with closed footwear.

## DISCUSSION

The World Health Organization defines diabetes as a chronic disease characterized by constantly elevated glycemia levels (exceeding 26 mg/dL) resulting from defects in insulin secretion and activity. These characteristic cause microvascular and macrovascular complications, including retinopathy, nephropathy, peripheral arterial disease, and ulcers of the lower limbs, known as the diabetic foot syndrome.^13^

The diabetic foot is defined as infection, ulceration, and/or destruction of deep tissues associated with neurological abnormalities and peripheral vascular disease.^6^ Neurological disease is an important risk factor for development of ulceration. Peripheral sensory-motor neuropathy and autonomic neuropathy are among the most common forms of neurological manifestation.^14^^,^ 15

The size of the sample chosen for this study was the result of chance, since we could not base the number of patients to be interviewed on data in the literature. Additionally, only a small percentage of these diabetic patients are members of the patient groups that meet regularly at the PHCCs for assessments and instructions.

In a study of 109 people from Rio Branco, AC, Brazil, educational level was not relevant in relation to knowledge about care to prevent the diabetic foot,^11^ probably because even people with higher levels of education do not have access to information on neuropathies and vascular disease.

Changes in sensitivity to pain are harmful because this is a protection against traumas or discomforts that could result in injury.^15^^,^ 16 Additionally, many physicians cannot understand how patients with a high degree of education can present at vascular surgery consultations with ulcers caused by shoes that are the wrong size. This can be explained by the fact that many patients already have some degree of neuropathy and shoes of a smaller size stimulate the remnant of sensitivity that is still present, making them conclude that the shoes are the correct size.^15^ This is not, therefore, a problem of an intellectual nature, but one of nerve damage.

The questions about going barefoot and wearing socks with closed shoes are aimed to identify habits that involve risk of development of ulceration. A study conducted in the city of Campinas, SP, Brazil, reported similar percentages with relation to wearing socks with shoes; but the residents of Campinas were more likely to go barefoot (36%)^12^ than the people from Maringá analyzed in our study (12.5%). However, the considerable percentage of illiterate participants (18.8%) in the study in Campinas led the authors to call attention to the need to create different educational strategies for this population that could facilitate self-care.^12^

In our study, 43.8% replied in the affirmative when asked if they wore socks with closed shoes, which is midway between two other studies, which reported figures of 57% and 37.5%, respectively.^12^^,^ 13 The quality of socks is very important. They should be made from wool or cotton, without seams, and should be changed every day, avoiding traumas caused by shoes.^17^

Appropriate footwear should support feet and protect them against mechanical traumas, distributing pressure points. They should not have stitching and should be in good condition. Shoes should not be too loose nor too tight, because both make friction and creation of blisters more likely, and they should ideally be bought during the afternoon, when the feet tend to have edema.^17^ Our study found that almost all of the interviewees were unaware of these details and only prioritized comfort when buying footwear.

While educational level does not have a direct relationship with diabetic ulcers, other factors such as visual deficiencies, poor balance, and loss of flexibility in the limbs limit the capacity to recognize abnormalities in the feet.^18^

Diabetic foot is related to duration of diabetes and patient age.^18^ The lifelong incidence of foot ulcers among diabetic patients is estimated at 12 to 25%.^19^ It is estimated that more than 50% of older patients with type 2 diabetes exhibit some type of evidence of sensory loss at clinical examination, a risk factor for ulceration, and that 13% of patients have relevant sensory loss at the time of diabetes diagnosis.^15^ These numbers show that periodic examinations of the feet of diabetic patients of any age, particularly in primary care, would result in early detection of significant neuropathic abnormalities, offering opportunities for reinforcement of therapeutic behavior and providing information on self-care.

In studies by Santos et al.^16^ and Gamba et al.,^20^ the majority of people who underwent amputations had poor metabolic control, did not have access to information on preventative care, did not comply with clinical treatment, and had financial difficulties. Furthermore, amputation and limb loss have a greater impact than any other complication of diabetes because, in addition to loss of mobility and independence, they frequently lead to anxiety and depression.^21^^,^ 22

Smoking is an aggravating cofactor that accelerates the process of atherosclerosis in tibial arteries.^23^ This, in addition to trauma, inappropriate footwear, foreign bodies in the feet, and incorrect nail cutting techniques, contributes to increasing the frequency of the diabetic foot.^11^ This factor should be given due weight, since around 20% of the sample in the present study continued smoking, despite being aware of the risks.

Care for the diabetic foot improves as patients acquire a clearer understanding of the factors that lead to limb loss and as a growing consensus is reached on the various different measures that need to be taken in relation to the feet.^4^^,^ 24

Programs run by the Brazilian Ministry of Health to care for patients with diabetes are based on the assumption that routine follow-up of patients will be managed through the structural axes of the PHCCs and the Family Health Strategy.^16^ However, there is no evaluation of the results of these programs with respect to preventative measures for the diabetic foot.

This study has observed that there are failures related to the primary care groups dedicated to providing instruction and care to hypertensive and diabetic patients. These HIPERDIA groups are apparently unsatisfactory in their fulfillment of the Ministry of Health’s recommendations, failing to achieve the principal purpose of primary care, which is to continuously monitor patients with hypertension and diabetes, both their conditions and their risk factors.^25^

It is nurses’ responsibility to provide guidance on lifestyle changes and to evaluate patients’ potential for self-care, in addition to assessing other risk factors, such as socioeconomic condition and educational level.^25^ However, a study conducted in Caxias, MA, Brazil, concluded that community health workers establish more effective links and are more effective at providing guidance,^26^ which, subjectively, has also been perceived by the authors of the present study.

We believe that it is the responsibility of the Ministry of Health to take educational measures, such as conducting visits and distributing pamphlets, designed to raise awareness among the diabetic population, since adequate glycemic control combined with healthy lifestyle habits and regular consultations targeting these clients could reduce the incidence of complications. Nevertheless, there is a lack of published data proving that these measures would be effective for complications of the diabetic foot.

Although some studies analyzed in a systematic review by the Cochrane Institute concluded that greater knowledge about preventative measures would not affect the incidence of diabetic foot, the same review concluded that the evidence in these studies is weak and based on methodology that is inadequate to rule out providing recommendations to patients with diabetes in practice.^27^

After the interviews, we informed the patients who participated in the study about self-care measures and gave them tips on prevention in educational pamphlets and also face-to-face, hoping to contribute to reducing the complications of their disease.

## CONCLUSIONS

There is a lack of knowledge about preventative measures, even among patients with a reasonable level of education, related to possible complications affecting the feet of diabetic patients.

Furthermore, higher monthly incomes were not reflected in greater interest in healthy lifestyle habits, supporting the conclusion that self-care is also neglected.
